# Interaction between neonatal maternal deprivation and serum leptin levels on metabolism, pubertal development, and sexual behavior in male and female rats

**DOI:** 10.1186/s13293-015-0054-6

**Published:** 2016-01-11

**Authors:** Virginia Mela, Francisca Díaz, María Jesús Vázquez, Jesús Argente, Manuel Tena-Sempere, Maria-Paz Viveros, Julie A. Chowen

**Affiliations:** Department of Physiology (Animal Physiology II), Faculty of Biology. Complutense University Madrid, Madrid, Spain; Department of Endocrinology, Hospital Infantil Universitario Niño Jesús, Instituto de Investigación la Princesa, Avenida Menéndez Pelayo, 65, Madrid, 28009 Spain; CIBEROBN, Instituto Carlos III Madrid, Madrid, Spain; Department of Cell Biology, Physiology and Immunology, University of Cordoba & Instituto Maimónides de Investigación Biomédica (IMIBIC), Hospital Universitario Reina Sofia, Córdoba, Spain; Department of Pediatrics, Universidad Autónoma de Madrid, Madrid, Spain

**Keywords:** Neonatal leptin surge, Sexual dimorphism, Neuropeptides, Hypothalamus, Puberty, Reproduction, Weight gain

## Abstract

**Background:**

Maternal deprivation (MD) during neonatal life can have long-term effects on metabolism and behavior, with males and females responding differently. We previously reported that MD during 24 h at postnatal day (PND) 9 blocks the physiological neonatal leptin surge in both sexes. It is known that modifications in neonatal leptin levels can affect metabolism in adulthood. Thus, we hypothesized that at least some of the long-term metabolic changes that occur in response to MD are due to the decline in serum leptin during this critical period of development. Hence, we predicted that treatment with leptin during MD would normalize these metabolic changes, with this response also differing between the sexes.

**Methods:**

MD was carried-out in Wistar rats for 24 h on PND9. Control and MD rats of both sexes were treated from PND 9 to 13 with leptin (3 mg/kg/day sc) or vehicle. Weight gain, food intake, glucose tolerance, and pubertal onset were monitored. Sexual behavior was analyzed in males. Rats were killed at PND90, and serum hormones and hypothalamic neuropeptides involved in metabolic control and reproduction were measured. Results were analyzed by three-way analysis of covariance using sex, MD, and leptin treatment as factors and litter as the covariate and employing repeated measures where appropriate.

**Results:**

In males, MD advanced the external signs of puberty and increased serum insulin and triglyceride levels and hypothalamic proopiomelanocortin mRNA levels at PND90. Neonatal leptin treatment normalized these effects. In contrast, MD decreased circulating triglycerides, as well as estradiol levels, in females at PND90 and these changes were also normalized by neonatal leptin treatment. Neonatal leptin treatment also had long-term effects in control rats as it advanced the external signs of puberty in control males, but delayed them in females. Neonatal leptin treatment increased serum insulin and hypothalamic mRNA levels of the leptin receptor and cocaine- and amphetamine-regulated transcript in control males and increased orexin mRNA levels in controls of both sexes. Although pubertal onset in males was advanced by either MD or neonatal leptin treatment in males and delayed by leptin treatment in females, the mRNA levels of hypothalamic neuropeptides and receptors related to reproduction were not affected by MD or neonatal leptin treatment in either sex at PND90.

**Conclusions:**

These findings indicate that some of the long-term changes in metabolic and reproductive parameters induced by MD, such as advanced pubertal onset and increased hypothalamic proopiomelanocortin (POMC) expression, hyperinsulinemia, and hypertriglyceridemia in adult males and decreased serum triglyceride and estradiol levels in females, are most likely due to the decrease in leptin levels during the period of MD.

## Background

Numerous studies have investigated the long-term impact of early stress in attempt to understand how the environment during development affects later health and well-being [1–4]. As hypothalamic development in rodents is not complete until the adolescent period, early environmental stress could disrupt the maturation of this brain region, which coordinates and controls endocrine systems [5]. This aberrant development could in turn induce long-term effects on metabolism and other neuroendocrine systems.

Many growth factors, metabolites, and hormones impact on brain development, with some of their effects being characteristic of specific brain nuclei or systems; for example, leptin is implicated in the development of metabolic circuits [6–8]. Leptin, an adipokine produced mainly by adipocytes, is involved in postnatal metabolic control [8–10]. It has also been implicated in hypothalamic development, as well as synaptic plasticity, neurogenesis, and neuronal and glial survival in diverse brain areas [6, 7, 10, 11]. In rodents, there is a postnatal leptin surge beginning around postnatal day (PND) 5 with a peak between PND9 and PND10 [12]. Some of the long-term metabolic outcomes in response to changes in neonatal nutrition are at least partially due to structural and functional changes in the hypothalamus that most likely result from the modifications of this leptin surge [8, 13, 14]. Leptin is also involved in reproductive function [15, 16], but the long-term effects of changes in the neonatal leptin surge on this system are less well studied.

During the last decade, we have studied maternal deprivation (MD) as a model of a psychoneuroimmunoendocrine disorder [17]. In these studies, we observed that MD during 24 h starting on PND9 induces long-term modifications in the endocrine, immune, endocannabinoid, and glutamatergic systems, as well as behavioral modifications, with many of these effects being sexually dimorphic [17]. During MD, there are important alterations in neuronal and glial markers and neurotrophic factors in the hypothalamus, such as a decrease in levels of nestin, brain-derived neurotrophic factor, and glial fibrillary acidic protein, with these modifications being generally more pronounced in males [18]. At PND13, 3 days after termination of the MD procedure, hypothalamic cell turnover is elevated, again with males being more affected [19]. These observations suggest that MD induces structural modifications in this brain area and that these modifications may be sexually dimorphic. During the 24-h MD protocol, the neonates experience a rise in serum corticosterone and a decrease in glucose, insulin, and leptin levels [18]. These changes are all potentially capable of inducing long-term effects on brain development [6, 20–23].

Growth and metabolism are affected by MD, with this experimental protocol resulting in reduced body mass, energy intake and circulating leptin levels throughout life [13]; however, these animals have an increased weight gain and inflammatory response to high-fat diet intake, as well as a sexually dimorphic response in changes in insulin sensitivity and metabolic hypothalamic neuropeptide expression [13]. Moreover, we have shown that blockage of the neonatal leptin surge not only affects long-term metabolism and the hypothalamic expression of metabolic neuropeptides in a sexually dimorphic manner [24], but also modulates the expression of members of the kisspeptin system in peripubertal rats [25], suggesting a possible effect on pubertal development. Hence, MD on PND9 could also affect pubertal development and reproductive function, possibly due to the dramatic decline in circulating leptin levels during a critical period of hypothalamic development.

Thus, the aims of this study were to: (1) delineate which of the previously described long-term metabolic/neuroendocrine effects of MD result from the MD-induced decline in circulating leptin levels; (2) determine if the MD procedure modifies the reproductive axis including the timing of pubertal onset, expression of gonadotrophins and neuropeptides involved in reproduction in the adult rat and male sexual behavior; (3) analyze whether the effects of MD on the reproductive axis are due to the reduction in circulating leptin levels that occurs during this procedure; (4) determine if males and females respond similarly to these experimental manipulations. To this end, rats subjected to MD and control rats received daily leptin treatment or vehicle between PND9 and PND13. Metabolic parameters, pubertal onset, sexual behavior, and hypothalamic metabolic and reproductive neuropeptide expression were analyzed to determine if this leptin treatment could protect from or palliate some of the deleterious effects of MD.

## Methods

### Animals

Adult Wistar rats were purchased from Harlan Interfauna Ibérica S.A. (Barcelona, Spain) and allowed to acclimate for 2 weeks before mating. For mating, one male was placed in a cage with two females for 10 days. On the day of birth (PND0), litters were culled to four male and four female pups per dam, with no cross-fostering being employed. In order to reduce the litter effect, three different litters were used in all experimental groups and in all analyses, with a total of 12 rats in each experimental group. Rats were maintained at a constant temperature (22 ± 1 °C) and humidity (50 ± 2 %) in a reversed 12-h light-dark cycle (red light on at 08:00 and white light on at 20:00) and given free access to rat chow (commercial diet for rodents 2918; Harlan Laboratories, Madison, WI, USA) and water.

The experimental procedures were approved by the University Ethical Committee for Animal Experimentation and complied with the Royal Decree 53/2013 and with the European Union guidelines for use of experimental animals (2010/63/EU).

### Maternal deprivation

**Fig. 1:**
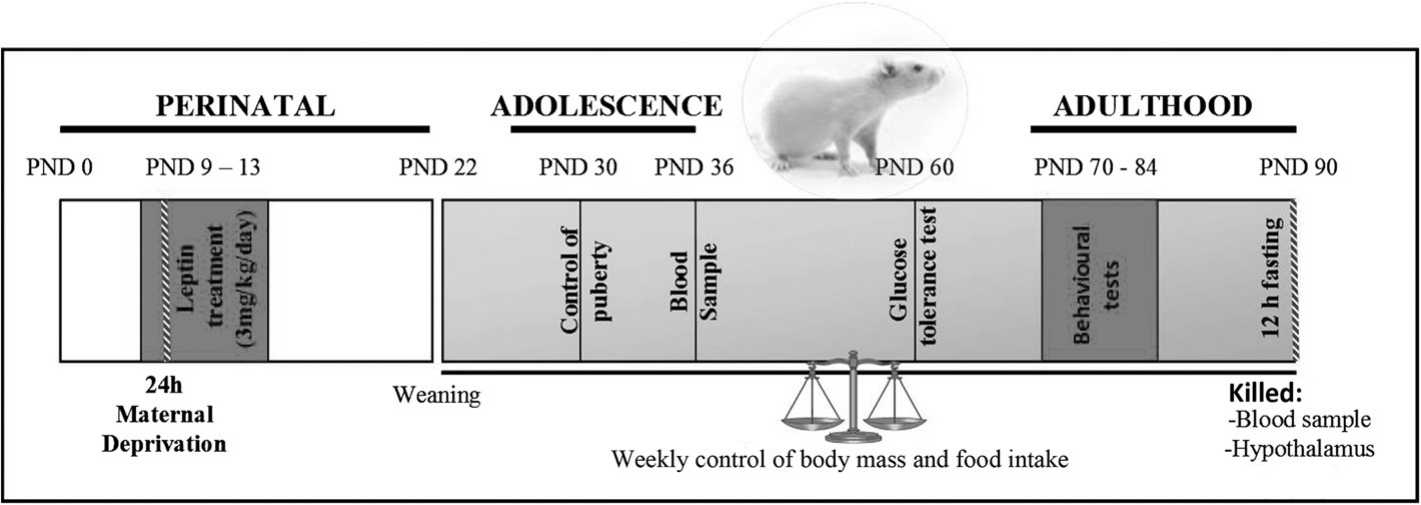
Experimental design diagram. This diagram indicates the day or days when maternal deprivation, leptin treatment, weaning, control of pubertal onset, blood sampling, glucose tolerance testing, and behavioral testing were performed. *PND* postnatal day

A schematic drawing of the experimental design is shown in Fig. 1. Maternal deprivation was performed as previously described [26]. Briefly, beginning at 09:00 on PND9, mothers from the deprived group were removed and placed in a cage next to the home cage in the same room and returned to their home cage on PND10. Mothers of the control litters were left undisturbed.

### Leptin treatment

Rats were injected sc with 3 mg/kg body mass (bm) of rat leptin (National Hormone and Pituitary Program, Torrance, CA) from PND9 until PND13. The rats received one injection per day at 9:00. Control rats received the same volume (2.5 ml/kg bm) of vehicle (saline + 0.1 % BSA). This leptin dose was selected as it has been previously shown to activate leptin signaling and modulate neuropeptide expression in the neonatal animal [27]. Because leptin returns to baseline levels 24 h after injection [28], leptin was administered daily. Daily injection was performed from the time of separation from their mother (PND9), until PND13 when the physiological neonatal leptin surge terminates [12]. After each injection, the rats were immediately returned to their mothers. All rats were then left undisturbed from PND13 until they were weaned at PND22. At weaning, the rats were separated and placed two rats of the same sex and experimental group/cage.

This treatment protocol resulted in four different groups of both males and females: Controls + vehicle: CoVh, Controls + leptin treatment: CoLeptin, maternal deprived + vehicle: MDVh, and maternal deprived + leptin treatment: MDLeptin. Each experimental group consisted of 12 animals from three different litters (4 animals/litter).

### Weight, food, pubertal control, and oral glucose tolerance test (OGTT)

Body mass and food intake were monitored weekly from weaning (PND22) until 5 days before being killed. Food intake was measured by placing a known amount of food in each cage and measuring the remaining amount at the same time the following week.

Pubertal onset was measured by monitoring vaginal opening (VO) or balano preputial separation (BPS). Due to the differences in age of pubertal onset, this monitoring started on PND30 in females and PND35 in males.

On PND60 at 9:00, an OGTT was performed. Glucose was administered orally (1 g/kg). Glucose levels were then determined in tail blood 15, 30, 60, and 120 min later by using a glucometer (Optium Plus, Abbot Diabetes Care, Witney Oxon, UK).

### Sexual behavioral testing

Female sexual behavior was not analyzed in order to avoid stress due to estrous cycle monitoring and to avoid the possibility of pregnancy.

Male sexual behavior was tested at PND84. For this test, stimulus female rats (300 ± 20 g) that were not a part of this study were induced to become sexually receptive by a subcutaneous injection of 50 mg of estradiol benzoate (Sigma-Aldrich) 48 h before testing, followed by injection of 500 mg of progesterone (Sigma-Aldrich) 4 h before testing. The experiments, performed during the central part of the dark period (12:00–16:00), were carried out in a square arena (60 cm × 60 cm × 45 cm) with matte-painted metallic floor and Perspex walls under red illumination provided by two 40 W fluorescent lamps. Each male rat was observed alone for 5 min. An estrous female was then introduced into the center of the arena and his behavior was recorded. Each test lasted a maximum of 30 min. If no intromission was displayed within the first 15 min, the test was terminated and the male was considered a “non-copulator”. Video tape recordings were later replayed and analyzed (in slow motion when necessary), and the following parameters were measured: (1) mount latency (ML; in seconds) defined as the time between introduction of the female rat and the first mount; (2) intromission latency (IL; in seconds) considered as the time between introduction of the female rat and the first intromission; (3) mount frequency, which was the number of mounts displayed before ejaculation; (4) intromission frequency, determined as the number of intromissions displayed before ejaculation; (5) ejaculation frequency, defined as the number of ejaculations during the test session; (6) ejaculation latency, which was the time between the first intromission and ejaculation; (7) total mounts/intromission frequency, calculated as the total number of mounts/intromission frequency; (8) inter-copulatory interval (ICI) calculated from the ejaculation latency/intromission frequency in each copulatory series; (9) pseudo inter-copulatory interval (pseudo-ICI) for non-ejaculating rats, which represents the time from the first intromission to the end of the 30 min session/number of intromissions; (10) copulatory rate (the sum of mounts and intromissions divided by the elapsed time (s) from the first mount until an observed ejaculation); (11) percentage of rats displaying copulatory activity; and (12) percentage of rats achieving ejaculation.

After each trial, animals were placed back into their home cages with littermates and the arena was thoroughly cleansed with water. These animals were submitted to other behavioral tests prior to the sexual test such as novel object test and elevated plus maze (manuscript submitted) with an interval of 7 days between each test.

For the statistical analyses, we used between 10 and 11 males and females per experimental group from three different litters (3–4 animals/litter/experimental group). All animals were submitted to the behavioral tests, and only the animals that did not fulfill the test requirements (two CoVh males, one CoLeptin male, one MDLeptin male, and two MDLeptin males) were excluded from the statistical analyses.

### Tissue collection

Blood was collected from the tail vein at PND36 in tubes containing EDTA (0.5 M) and rapidly placed on ice. On PND90, after a 12-h fast, all rats were killed by rapid decapitation. The estrous stage of each female was determined on the day of sacrifice, with 80 % of the rats found to be in estrus and 20 % in metestrus, with no difference between groups. Trunk blood was collected in tubes and rapidly placed on ice and kept overnight at 4 °C. The blood was centrifuged (1600*g* for 15 min) and the serum collected and stored at −20 °C until processed. The brain was rapidly removed and the hypothalamus was dissected and the pituitary was collected. They were then frozen in liquid nitrogen and stored at −80 °C until processed.

### Leptin enzyme-linked immunosorbent assay (ELISA)

Plasma leptin levels were measured at PND36 by using B-bridge mouse/rat leptin ELISA kits (Cupertino, CA, USA) following the manufacturer’s instructions. The assay sensitivity is 0.5 ng/ml, with an inter-assay variation of 6.5 % and intra-assay variation of 3.7 %. All samples were run in duplicate. For this assay, we used 6 males and 5–6 females per experimental group from three different litters (1–2 randomly selected animals/litter/experimental group).

### Multiplexed magnetic bead immunoassay

Insulin levels were measured in duplicate in serum collected at PND90 by a multiplexed magnetic bead immunoassay kit (Millipore Corporation) as previously described [29]. Briefly, beads conjugated to the appropriate antibodies and serum samples (25 μl each) were incubated overnight at 4 °C with shaking. Wells were washed three times using a wash buffer and antibody conjugated to biotin (50 μl) was added. After incubation for 30 min at room temperature with shaking, beads were incubated during 30 min with 50 μl streptavidin conjugated to phycoerythrin. Beads were analyzed in the Bio-Plex suspension array system 200. Raw data (mean fluorescence intensity) was analyzed using the Bio-Plex Manager Software 4.1 (Bio-Rad Laboratories). Leptin was also measured in this assay. These results have been previously reported (manuscript submitted).

For this analysis, we used between 5 and 8 males and females per experimental group from three different litters (1–3 randomly selected animals/litter/experimental group).

### Corticosterone and testosterone radioimmunoassay

Corticosterone was measured in serum collected at PND36 and PND90 using a solid phase I125 radioimmunoassay (Immunochem Double Antibody Cort; MP Biomedicals, Illkirch, France). The detection limit was 7.7 ng/ml and the inter- and intra-assay coefficients of variation were less than 10 %.

Plasma testosterone levels were measured in serum collected at PND90 with RIA Coat-a-Count® kits from Siemens (Los Angeles, USA) following the manufacturer’s instructions. The detection level was 4 ng/dl. The intra- and inter-assay coefficients of variation were 6.0 and 6.4 %, respectively.

Between 5 and 8 males and females per experimental group from three different litters (1–3 randomly selected animals/litter/experimental group) were used for these assays.

### Estradiol-17β ELISA

Plasma 17-β estradiol levels were determined in serum collected at PND90 by using an ELISA from Enzo (Farmingdale, NY, USA). Absorbance in each well was measured by using a Tecan Infinite M200 (Grodig, Austria), and serum concentrations were calculated from the standard curve. This assay has a sensitivity of 14.0 pg/ml and inter- and intra-assay coefficients of variation of 8.3 and 2.1 %, respectively.

All samples were run in duplicate and in the same assay for all hormones. For this assay, between 5 and 6 females per experimental group from three different litters (1–2 randomly selected animals/litter/experimental group) were used.

### Triglyceride assay

Triglycerides were measured in serum collected at PND90 by using commercial kits (Randox, Antrim, UK) according to the manufacturer’s instructions. Serum samples (in duplicate) were incubated with the commercial solution containing lipoprotein lipase, glycerol kinase, glycerol-3-oxidase, peroxidase, 4-aminophenazone, and ATP for 5 min at 37 °C under agitation. The resulting color was detected at 500 nm in a spectrophotometer and compared to a standard curve.

For this assay, we used between 7 and 8 males and females per experimental group from three different litters (2–3 animals/litter/experimental group, randomly selected).

### Quantitative real-time PCR

Total RNA from approximately 50–100 mg of hypothalamic tissue, divided into rostral (HTr) and caudal or medial basal (MBH) areas using the optic chiasm as reference, was isolated using TRIsure Reagent (Bioline, London). High-Capacity cDNA Reverse Transcription kits (BioRad Laboratories, Hercules, CA) were used according to the manufacturer’s protocol on a iCycler iQ PCR Thermal Cycler (BioRad) to transcribe 0.5 μg total RNA isolated from each tissue.

Aliquots of the resulting complementary deoxyribonucleic acid (cDNA) samples were amplified by PCR with specific oligonucleotide primer pairs designed to span intron/exon borders. The sequence of the primer pairs used were [1] rat Kiss1, forward 5′ GCT GCT GCT TCT CCT CTG TG 3′ and reverse 5′GCA TAC CGC GGG CCC TTT T 3′; [2] rat Gpr54, forward 5′GCC ACA GAT GTC ACT TTC CTT C 3′ and reverse 5′GCC ACA GAT GTC ACT TTC CTT C 3′; [3] rat RFRP (RFamide-related peptide), forward 5′ AAT CCC TGC ACT CCC TGG CCT 3′ and reverse 5′AAG GAC TGG CTG GAG GTT TCC 3′; and [4] rat NPFFR1 (neuropeptide FF receptor 1), forward 5′ AAC CGG CAC ATG CGC ACT GTC 3′and reverse 5′ GAC ATG CCC TGC ACC AAG CCG 3′. Amplification of S11, forward 5′ CAT TCA GAC GGA GCG TGC TTA C 3′ and reverse 5′ TGC ATC TTC ATC TTC GTC AC 3′ served as control for the RT-PCR reactions. Amplification of the cDNA template was performed using 1x iQ Supermix and SYBR green for each detected gene (Promega, Wisconsin, USA). Predesigned commercial expression assays were used for the following genes: neuropeptide Y (NPY; Rn01410145_m1), proopiomelanocortin (POMC; Rn00595020), agouti-related peptide (AgRP; Rn014311703_g1), cocaine- and amphetamine-regulated transcript (CART; Rn00567382_m1), orexin (Rn00565995_m1) and the leptin receptor (LepR; Rn01433250_m1). Results were normalized to Pgk1 (Rn00569117_m1) mRNA levels in all samples.

Total RNA was extracted from each pituitary with TRIzol® Reagent (Invitrogen). High-Capacity cDNA Reverse Transcription kits (Applied Biosystems, Foster City, CA) were used according to the manufacturer’s protocol on a Peltier thermal Cycler Tetrad2 (BioRad) to transcribe 2 μg total RNA. Amplification of the cDNA template was performed with an ABI PRISM 7900HT sequence detection system (Applied Biosystems) using TaqMan Universal PCR Master Mix (Applied Biosystems) and TaqMan Gene Expression Assay kits for each gene (Applied Biosystems). The pituitary follicle-stimulating hormone (βFSH; Rn01484594_m1) and luteinizing hormone (βLH; Rn00563443_g1) were analyzed. Results were normalized to 18S (Rn01428915_g1) mRNA levels.

According to manufacturer’s guidelines, the ΔΔCT method was used for relative quantification. Statistics were performed using ΔCT values.

Between 5 and 6 males and females per experimental group from three different litters (1–2 randomly selected animals/litter/experimental group) were used for these assays. Samples that did not meet the standards of purity and/or integrity were excluded.

### Statistical analysis

Normality was checked by Shapiro-Wilks’s test (*p* > 0.05). The criterion of normally distributed data were not always met in some populations under study; thus, when necessary data were transformed by the Neperian logarithm function, aiming to satisfy the assumption of normality for ANOVA. To control for a possible litter effect, we performed ANCOVA analyses (including “litter” as a covariate). Body mass gain and food intake were analyzed by three-way ANCOVA with the factors being sex (males and females), MD (Co: maternal non-deprived; MD: deprived rats) and leptin treatment (Vh: vehicle; Leptin treatment: Leptin), with repeated measures. Three-way ANCOVA was used to analyze results when tissue from males and females were processed simultaneously (hormone assays, OTTG, and PCR), followed by two-way when appropriate. Two-way ANCOVAs were employed when males and females were processed separately (pubertal onset, sex hormone assays, and sexual behavior). Tukey’s test was used for post hoc comparisons. The level of significance was chosen as *p* < 0.05. All results are reported as mean ± SEM. In the figures, only physiologically relevant comparisons are demonstrated. Although changes in body mass were analyzed simultaneously in both sexes, the results are represented in the figures separately to facilitate interpretation.

## Results

### Effects of MD and leptin treatment on metabolic parameters: body mass gain, accumulated food intake, and OGTT

**Fig. 2:**
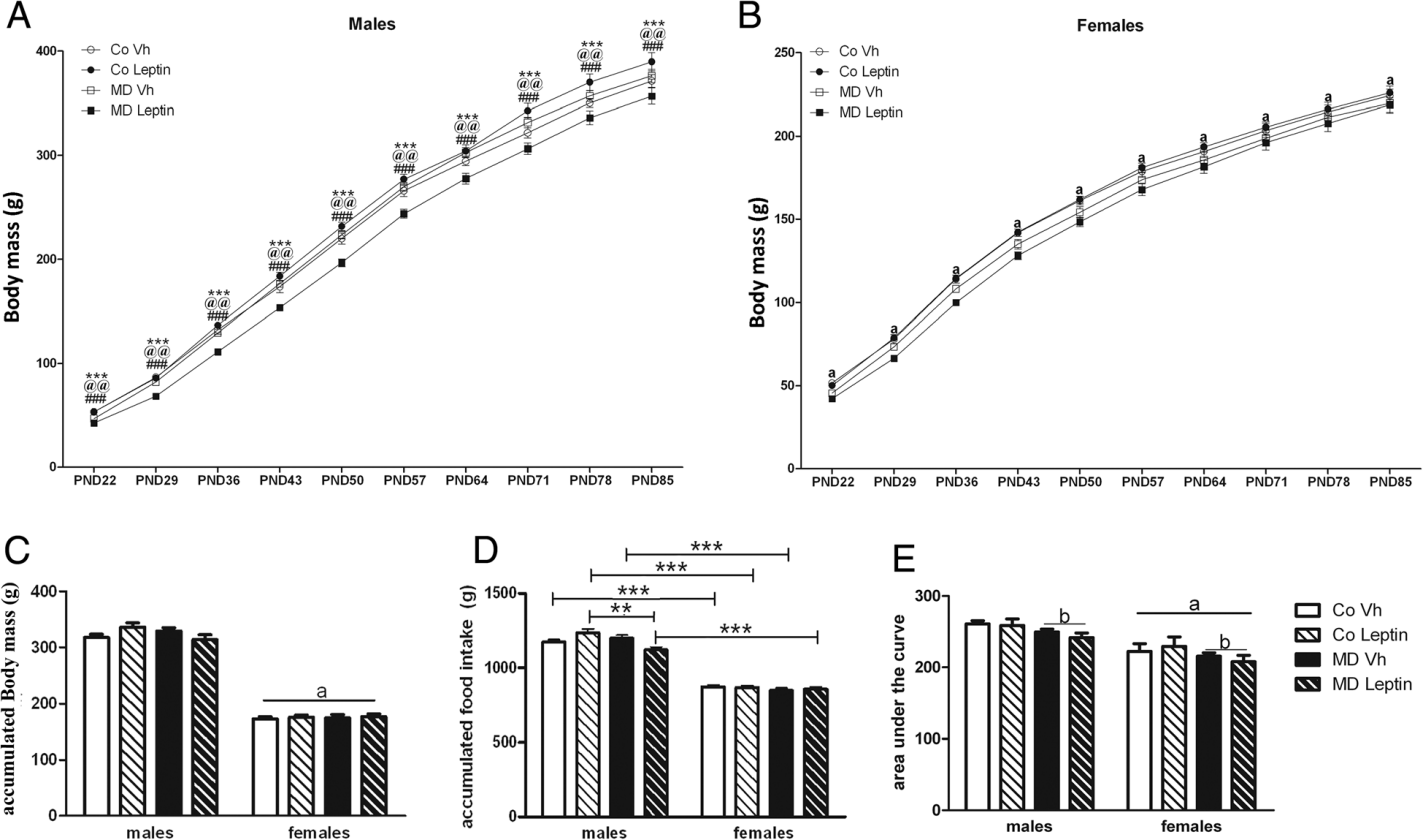
Changes in body mass and food intake throughout the study. **a**, **b** Mean body mass (BM), **c** accumulated BM gain, and **d** accumulated food intake per rat from the time of weaning until 5 days before being killed. **e** Glucose tolerance test (area under the curve), in maternal deprivation (MD) or control (Co) male and female rats treated with leptin (Lept) or vehicle (Vh) from postnatal day (PND) 9 until PND13 (*n* = 10–12). Body mass was reduced by MD in both sexes until approximately PND57, while leptin treatment of MD rats returned body mass to control levels. Males weighed more and ate more than females throughout the study and were less glucose tolerant. **a**, **b** Data were analyzed by a repeated measure three-way ANCOVA: (*a*) main effect of sex; Tukey’s post hoc test: *** CoLeptin vs. MDLeptin (*p* < 0.005), *@@* MDLeptin vs. CoVh (*p* < 0.01) and *###* MDVh vs. MDLeptin (*p* < 0.005). **c**–**e** Data were analyzed by three-way ANCOVA: (*a*) main effect of sex; (*b*) main effect of MD. Tukey’s post hoc: **p < 0.01; ***p < 0.005

Body mass varied according to age [*F*(2.07,729) = 1166.80; *p* < 0.005], sex [*F*(1,81) = 923.55; *p* < 0.005] with an interaction between age and sex [*F*(2.07,729) = 969.37; *p* < 0.005], with females weighing less than males, and to MD [*F*(1,81) = 22.36; *p* < 0.005] with an interaction between age × sex × MD × Lept [(2.07,729) = 4.01; *p* < 0.05]. MD rats of both sexes had a lower body mass than their controls until PND57 (Fig. 2a, b). A significant effect of sex [*F*(1,81) = 265.67; *p* < 0.005] was found on accumulated body mass gain with female rats gaining less than males (Fig. 2c).

There was an interaction between sex × MD × leptin [*F*(1,81) = 7.96; *p* < 0.01] on the accumulated energy intake. Females ate less than males in all corresponding groups (Fig. 2d). Leptin-treated MD males consumed less energy than CoVh males.

Significant effects of sex [*F*(1,81) = 45.81; *p* < 0.005] and neonatal leptin treatment [*F*(1,81) = 8.99; *p* < 0.01] were found on baseline glucose levels (males: CoVh 81.0 ± 2.8; CoLeptin 101.3 ± 3.8; MDVh 88.2 ± 2.2; MDLeptin 96.4 ± 1.8; females: CoVh 76.2 ± 2.9; CoLeptin 81.4 ± 3.3; MDVh 73.2 ± 3.8; MDLeptin 87.8 ± 5.3 mg/dl), with males having higher levels than in females and leptin treatment increasing baseline glucose levels. During the OGTT, there were significant effects due to sex [*F*(1,81) = 33.55; *p* < 0.005] and MD [F(1,81) = 4.33; *p* < 0.05]. Female rats were more glucose tolerant than males and MD increased glucose tolerance in both sexes (Fig. 2e), with no effect of leptin treatment.

### Long-term effect of MD and neonatal leptin treatment on circulating hormone and triglyceride levels

**Fig. 3:**
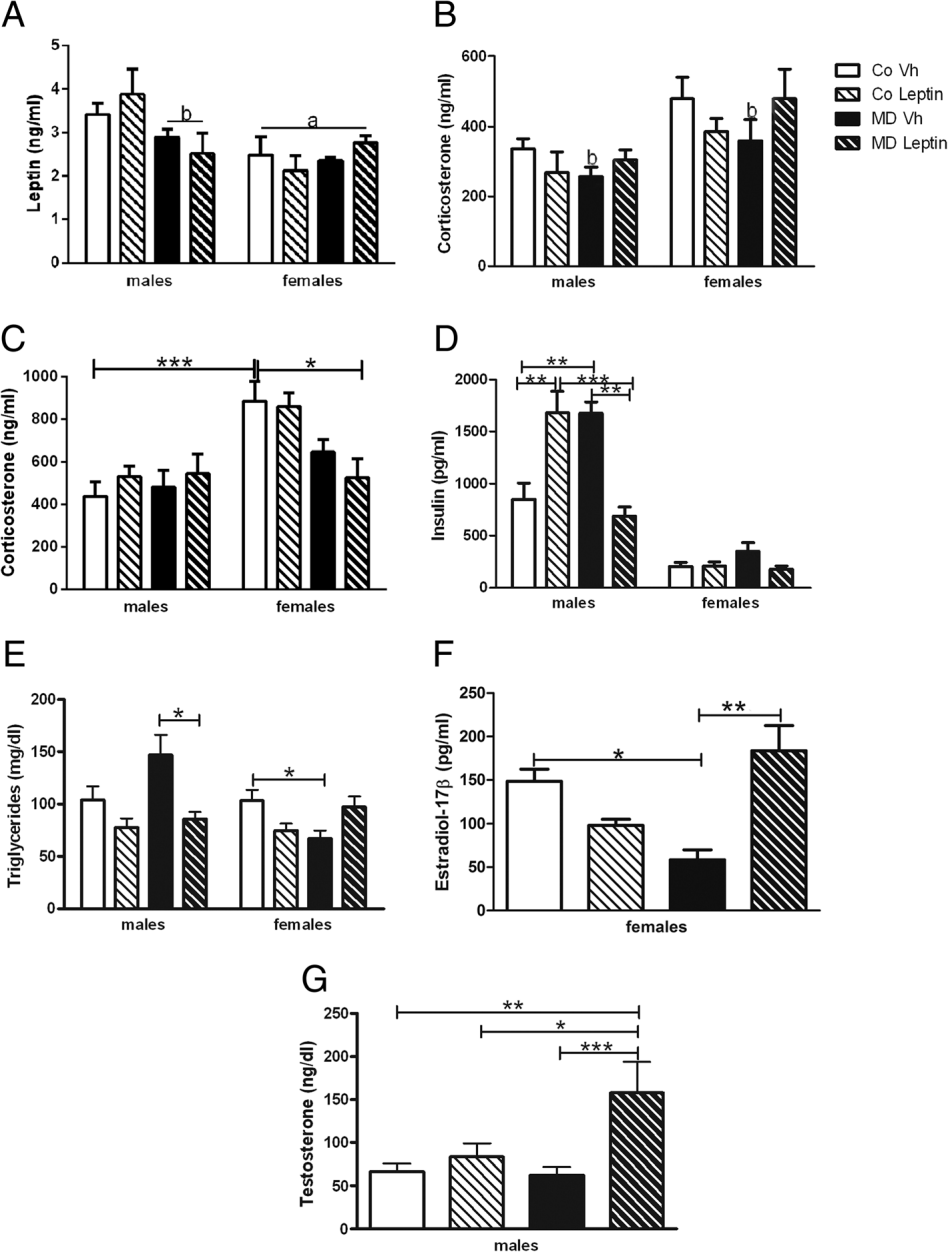
Serum levels of leptin (**a**) and corticosterone at postnatal day (=ND)36 (**b**) and of corticosterone (**c**), insulin (**d**), 17β-estradiol (**e**), and testosterone (**f**), at PND90 in maternal deprivation (MD) or control (Co) male and female rats treated with leptin (Lept) or vehicle (Vh) from PND9 until PND13 (*n* = 7–8). In males, MD decreased leptin and increased insulin and triglyceride levels, with leptin treatment returning insulin and triglycerides to control levels. In contrast in females, MD decreased triglyceride and estradiol levels, which were also normalized by leptin treatment. *a* Main effect of sex (three-way ANCOVA). *b* Main effect of MD (two-way ANCOVA). Tukey’s post hoc test:*p < 0.05; **p < 0.01; ***p < 0.005

At PND36, a main effect of sex [*F*(1,37) = 9.83; *p* < 0.005] was found on circulating leptin levels. Females had lower leptin levels (Fig. 3a) than males. An interaction between sex and MD was also found [*F*(1,37) = 4.32; *p* < 0.05] and two-way ANCOVA (split by sex) revealed a decrease in leptin levels in males due to MD [*F*(1,19) = 6.18; *p* < 0.05].

To determine if corticosterone levels remained elevated as seen during the MD protocol [17], serum levels were measured at PND36. A main effect of sex [*F*(1,32) = 12.51; *p* < 0.005] was found with females having higher corticosterone levels. An interaction between MD and leptin treatment was also found [*F*(1,32) = 5.05; *p* < 0.05], and two-way ANCOVA (split by leptin treatment) revealed a decrease in corticosterone levels in MDVh males [*F*(1,16) = 11.14; *p* < 0.005] and females [*F*(1,15) = 4.84; *p* < 0.05] (Fig. 3b).

At PND90, a significant effect of sex was found on circulating corticosterone [*F*(1,55) = 13.88; *p* < 0.005] levels. Females had higher corticosterone (Fig. 3c) levels than males regardless of treatment. An interaction between sex and leptin treatment was found [*F*(1,55) = 7.25; *p* < 0.01], with MDLeptin females having lower levels than CoVh females.

At PND90, there was an interaction between MD and leptin treatment on insulin levels in males [*F*(1,22) = 36.03; *p* < 0.005], with both CoLeptin males and MDVh males having higher levels than in CoVh males. Leptin treatment normalized the increase observed in MD males (Fig. 3d).

A main effect of leptin treatment [*F*(1,52) = 7.98; *p* < 0.01] was found on circulating triglyceride levels, with an interaction between sex, MD, and leptin treatment [*F*(1,52) = 7.03; *p* < 0.05]. Two-way ANCOVA (split by sex) revealed an effect of leptin treatment in males [*F*(1,25) = 14.34; *p* < 0.005] and an interaction between MD and leptin treatment in females [*F*(1,26) = 9.93; *p* < 0.005]. As shown in Fig. 3e, triglycerides levels tended to increase in MDVh males and leptin treatment counteract this effect (Tukey’s post hoc, *p* < 0.05), while females MDVh showed lower levels than in the CoVh group (Tukey’s post hoc, *p* < 0.05).

As we have previously found MD to modify sex steroid levels in adult males [19, 30], we analyzed sex steroid levels at PND90 to determine the effect of leptin treatment. There was an interaction between MD and leptin treatment on estradiol levels in PND90 females [*F*(1,16) = 13.21; *p* < 0.005], with MDVh females having lower levels than in CoVh females. This was normalized in MDLeptin females (Fig. 3f).

In males, there was an effect of leptin treatment on testosterone levels [*F*(1,22) = 6.84; *p* < 0.05] at PND90 with higher levels in MD males than the rest of the experimental groups (Fig. 3g).

### Hypothalamic mRNA levels of genes related to metabolic control

**Fig. 4:**
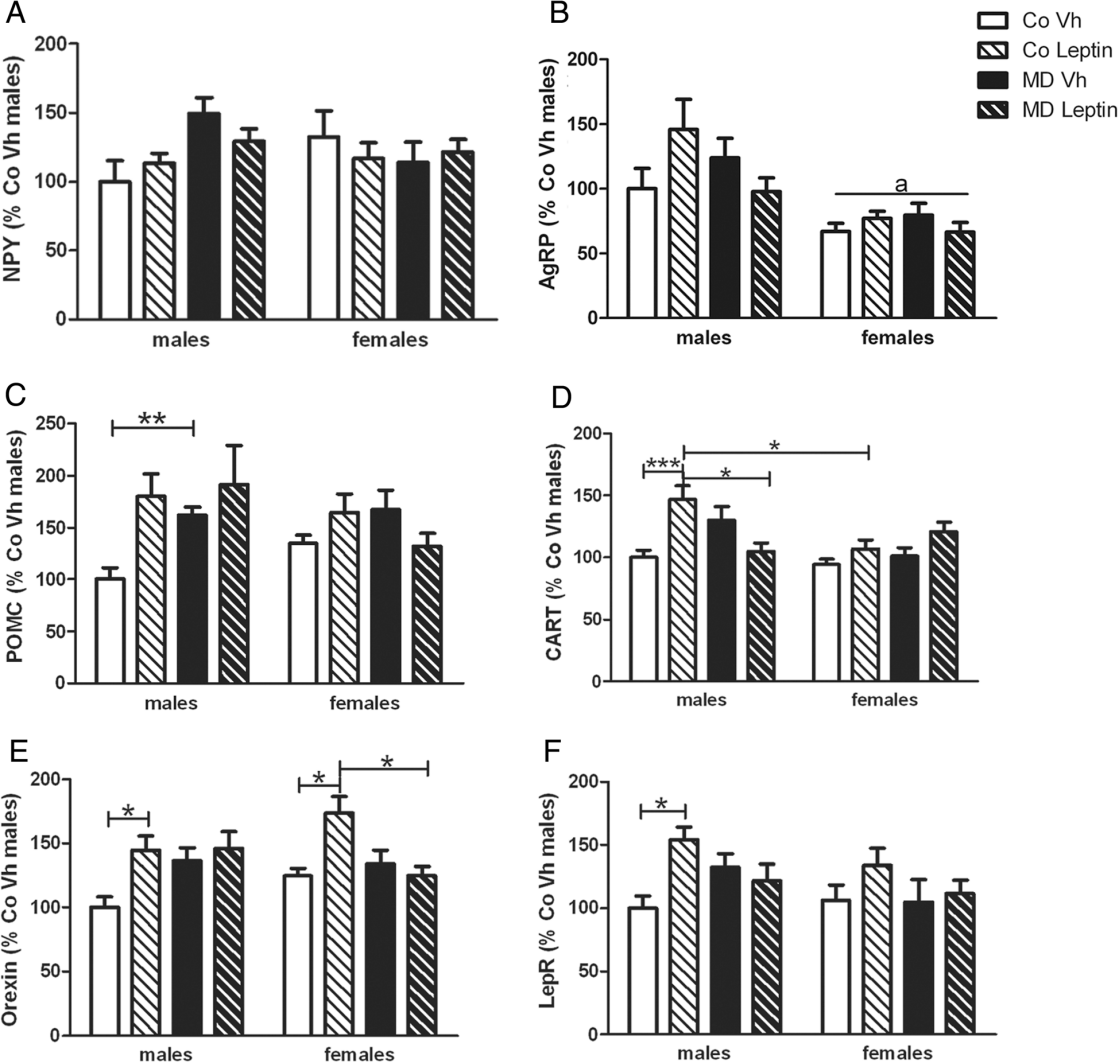
Analysis of hypothalamic neuropeptide mRNA levels. Expression levels in the medial basal hypothalamus of the mRNAs encoding NPY (**a**), AgRP (**b**), POMC (**c**), CART (**d**), orexin (**e**), and leptin receptor (LepR) (**f**) in maternal deprivation (MD) or control (Co) male and female rats treated with leptin (Lept) or vehicle (Vh) from postnatal day (PND) 9 until PND13 (*n* = 5–6). In males, MD increased POMC mRNA levels, but leptin treatment did not normalize this effect. In contrast, leptin treatment of control males increased CART, orexin, and LepR mRNA levels. In females, there was no effect of MD on the expression of these neuropeptides and leptin treatment of control females only increased orexin expression. Three-way ANCOVA: (*a*) main effect of sex. Tukey’s post hoc test: *p < 0.05; **p < 0.01; ***p < 0.005

Although we previously found no effect of MD on hypothalamic expression of neuropeptides involved in food intake in adult rats [13, 19], changes in the actions of neonatal leptin can affect these parameters [8, 25, 28]. As previously reported [13, 19], we found no effect of MD on NPY (Fig. 4a) or AgRP (Fig. 4b) mRNA levels in either sex. There was also no effect of neonatal leptin treatment. However, females had lower AgRP mRNA levels compared to males [*F*(1,36) = 19.80; *p* < 0.005].

There was an interaction between MD and leptin treatment on both POMC [*F*(1,37) = 6.83; *p* < 0.05] and CART [*F*(1,38) = 7.47; *p* < 0.01] mRNA levels. In males, POMC mRNA levels were increased by MD (Fig. 4c). Leptin increased CART mRNA levels in control males, but not in MD males (Fig. 4d). Although there was no difference in controls, leptin-treated control males had higher levels of CART mRNA than in leptin-treated control females.

There was an interaction between MD and leptin treatment [*F*(1,34) = 9.53; *p* < 0.005] on orexin mRNA levels in the MBH. Leptin treatment increased orexin mRNA levels in control males and females (Fig. 4e).

There was no effect of MD on LepR mRNA levels, but leptin treatment increased them in control males [*F*(1,35) = 7.90; *p* < 0.01] (Fig. 4f).

### Hypothalamic mRNA levels of genes related to reproduction

Females had significantly higher *Kiss1* mRNA levels in the HTr [*F*(1,43) = 88.38; *p* < 0.005] and in the MBH [*F*(1,42) = 28.49; *p* < 0.005] compared to males. However, no effects of leptin treatment or MD were found. There were no significant effects on the mRNA levels of any of the other factors or receptors involved in the neuroendocrine control of reproduction, including *Npff1r*, *Gpr54*, and *Rfrp,* in either hypothalamic area (Table 1).

**Table 1:** mRNA levels of puberty-related genes in rostral (HTr) and medial basal hypothalamus (MBH)

	Sex	CoVh	CoLept	MDVh	MDLept
*Kiss1* HTr	M	100 ± 8.0	95.6 ± 16.0	109.7 ± 9.0	114.4 ± 25.0
	F	438.4 ± 146.2^a^	467.1 ± 109.8^a^	506.8 ± 84.3^a^	319.2 ± 45.6^a^
*Kiss1* MBH	M	100 ± 8.0	126.3 ± 12.9	116.4 ± 8.7	128.0 ± 19.5
	F	192.0 ± 22.2^a^	185.5 ± 33.6^a^	172.1 ± 18.8^a^	215.6 ± 31.5^a^
*Gpar54* HTr	M	100 ± 9.2	95.4 ± 6.6	102.5 ± 6.3	92.2 ± 12.3
	F	123.0 ± 12.2	100.8 ± 6.3	100.8 ± 6.1	94.8 ± 4.9
*Gpar54* MBH	M	100 ± 8.0	99.4 ± 3.9	103.9 ± 5.0	111.9 ± 9.6
	F	119.9 ± 4.1	104.0 ± 5.5	112.6 ± 16.0	104.0 ± 7.3
*Rfrp* HTr	M	100 ± 43.6	17.0 ± 3.4	31.7 ± 6.4	39.8 ± 15.8
	F	49.3 ± 11.4	39.0 ± 12.3	88.6 ± 26.1	90.8 ± 38.7
*Rfrp* MBH	M	100 ± 12.9	107.8 ± 13.8	95.1 ± 10.8	117.2 ± 13.7
	F	104.9 ± 7.0	110.5 ± 18.6	112.0 ± 11.5	109.9 ± 6.0
*Nprffr1* HTr	M	100 ± 8.1	110.0 ± 5.4	100.1 ± 4.0	106.7 ± 8.9
	F	107.1 ± 11.2	119.7 ± 4.7	98.3 ± 5.6	94.0 ± 12.1
*Nprffr1* MBH	M	100 ± 12.8	129.2 ± 7.9	110.6 ± 8.2	115.9 ± 7.5
	F	107.4 ± 5.1	116.1 ± 10.7	100 ± 5.8	108.6 ± 15.4

Data are expressed as mean ± SEM and normalized to control (Co) values. Co and maternal deprivation (MD) male (M) and female (F) rats with leptin treatment (Lept) or vehicle (Vh) from postnatal day (PND) 9 to PND13. *n* = 5–6 per experimental group

^a^Main effect of sex; three-way ANOVA

#### Pituitary gonadotrophin mRNA levels

There was no effect of MD or neonatal leptin treatment on βFSH or βLH mRNA levels (Table 2). However, females had lower levels of βFSH mRNA in the pituitary than males [*F*(1,33) = 77.74; *p* < 0.005].

**Table 2:** Gonadotropin mRNA levels in the pituitary

	Sex	CoVh	CoLept	MDVh	MDLept
βFSH	M	100 ± 35.2	47.5 ± 11.7	67.7 ± 9.1	210.9 ± 74.0
	F	13.4 ± 4.9^a^	11.4 ± 5.0^a^	13.0 ± 6.7^a^	4.7 ± 0.8^a^
βLH	M	100 ± 34.2	82.2 ± 20.0	76.3 ± 8.0	170.8 ± 70.2
	F	86.7 ± 41.2	138.2 ± 50.4	69.4 ± 13.8	80.7 ± 9.2

Data are expressed as mean ± SEM and normalized to control (Co) values. Control and maternal deprivation (MD) male (M) and female (F) rats with leptin treatment (Lept) or vehicle (Vh) from postnatal day (PND) 9 to PND13. *n* = 5–6 per experimental group

^a^Main effect of sex; three-way ANOVA

#### Pubertal onset

**Fig. 5:**
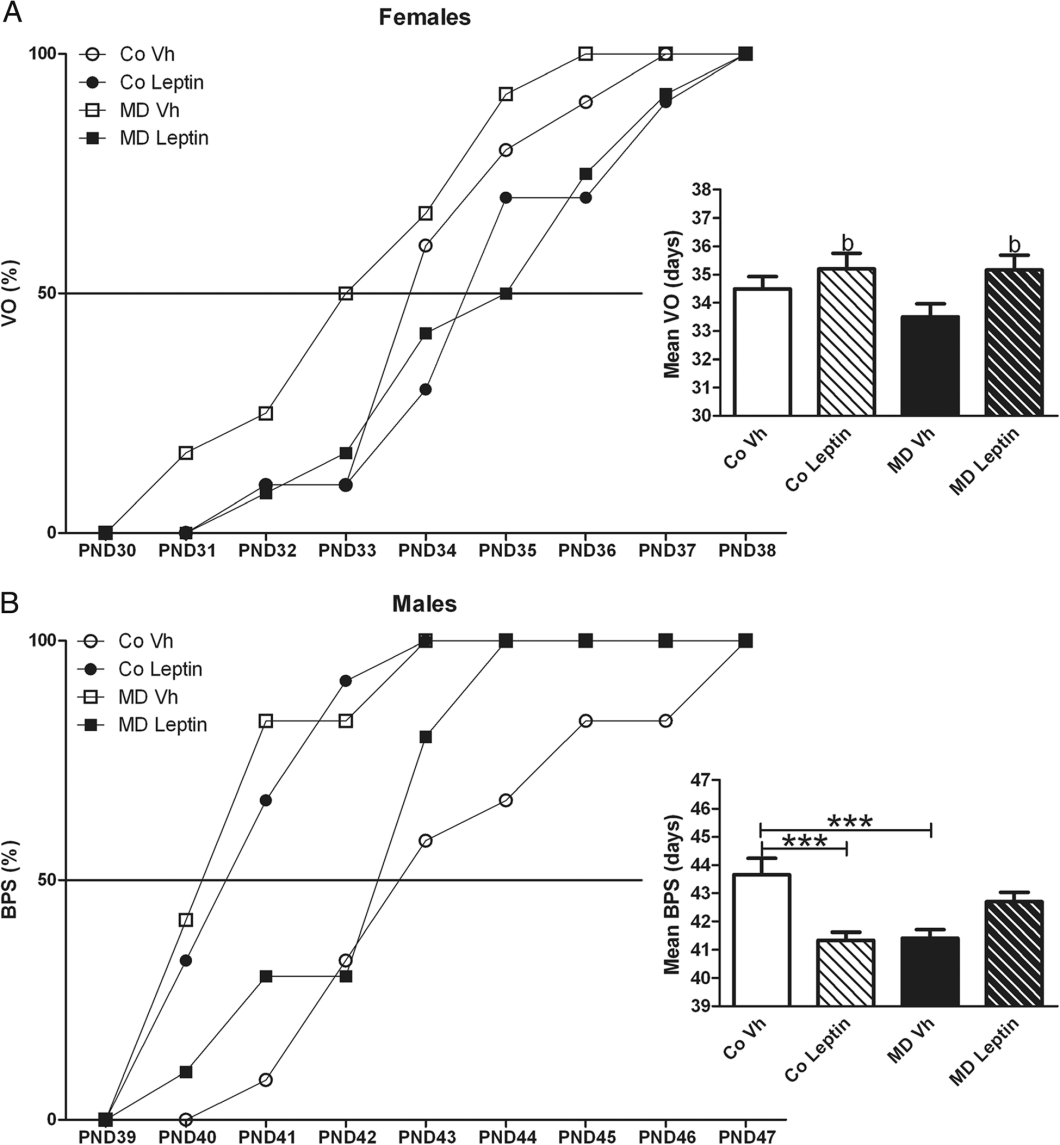
Effect of neonatal maternal deprivation and leptin treatment on pubertal onset. Age of pubertal onset assessed by vaginal opening (VO) in females (**a**) and balano-prepurcial separation (BPS) in males (**b**) in maternal deprivation (MD) or control (Co) rats treated with leptin (Lept) or vehicle (Vh) from postnatal day (PND) 9 until PND13 (*n* = 10–12). The animals were monitored between days 30 and 38 (females) or days 35 and 47 (males) for VO or BPS, respectively. In females, neonatal leptin treatment delayed the mean age of VO in both control and MD rats, with no effect of MD alone. In males, external pubertal signs were advanced with both MD and leptin treatment, with the combination of these factors normalizing pubertal onset. For the presentation of data, cumulative percentage data of VO/BPS are shown beside the mean age of VO/BPS. Two-way ANCOVA: (*b*) main effect of leptin treatment. Tukey’s post hoc: ***p < 0.005; *p < 0.05

There was no effect of MD on VO (Fig. 5a). However, neonatal leptin treatment delayed VO [*F*(1,39) = 6.23; *p* < 0.05] regardless of whether the females had been exposed to MD or not (Fig. 5a).

There was an interaction between MD and leptin treatment on BPS [*F*(1,41) = 23.92; *p* < 0.005]. MDVh males and CoLeptin males showed anticipated pubertal onset compared with CoVh males; however, leptin treatment of MD males normalized the appearance of external signs of pubertal onset (Fig. 5b).

### Sexual behavior

**Fig. 6:**
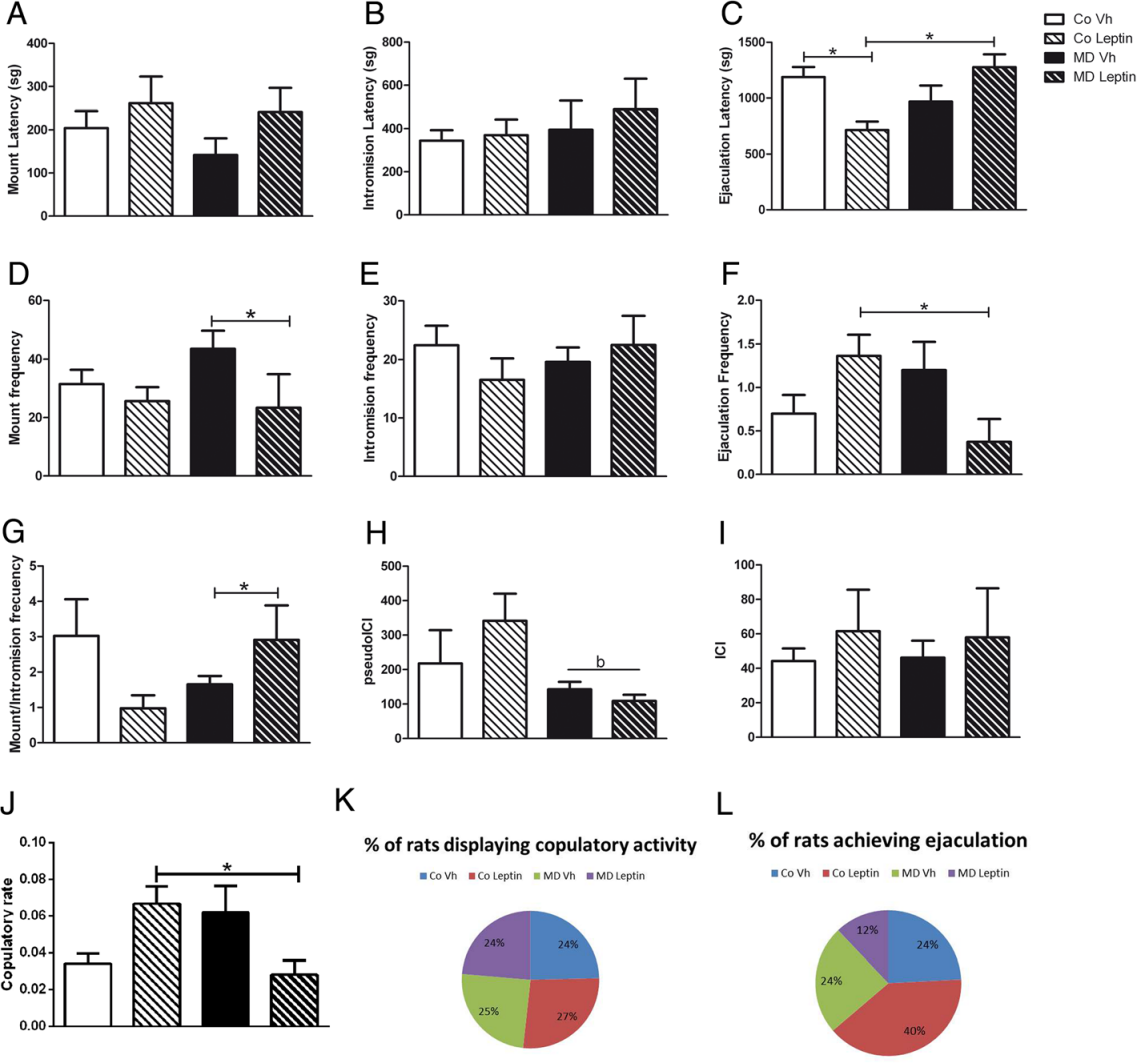
Sexual behavior in male rats. **a** Mount latency, **b** intromission latency, **c** ejaculation latency, **d** mount frequency, **e** intromission frequency, **f** ejaculation frequency, **g** mount/intromission frequency, **h** pseudo-ICI (pseudo intercopulatory interval for non-ejaculating rats), **i** ICI (inter-copulatory interval: ejaculation latency/intromission frequency in each copulatory series), **j** copulatory rate (the sum of mounts and intromissions divided by the elapsed time (s) from the first mount until an observed ejaculation), **k** percentage of rats displaying copulatory activity, and **l** percentage of rats achieving ejaculation, of maternal deprivation (MD) or control (Co) male and female rats treated with leptin (Lept) or vehicle (Vh) from postnatal day (PND) 9 until PND13 (*n* = 10–12). Ejaculation frequency was increased by leptin in control rats, but decreased by leptin in MD rats, with the inverse effect seen on ejaculation latency. Two-way ANCOVA: (*b*) main effect of MD. Tukey’s post hoc: *p < 0.05

Although leptin treatment tended to increase mount latency, this effect was not significant (Fig. 6a) and no effect was found on intromission latency (Fig. 6b). An interaction between MD and leptin treatment was found on ejaculation latency [*F*(1,34) = 14.41; *p* < 0.005] with neonatal leptin treatment decreasing ejaculation latency in control males but not in MD males. CoLeptin males had a lower ejaculation latency than MDLeptin males (Fig. 6c).

Neonatal leptin treatment tended to decrease mount frequency [*F*(1,35) = 4.08; *p* = 0.05; Fig. 6d] and to increase mount/intromission frequency [*F*(1,35) = 4.88; *p* < 0.05] in MD males (Fig. 6g). There was an interaction between MD and leptin treatment [*F*(1,35) = 6.22; *p* < 0.05] with leptin increasing ejaculation frequency in controls [*F*(1,21) = 7.16; *p* < 0.05] and decreasing it in MD rats [*F*(1,19) = 7.92; *p* < 0.05; Fig. 6f]. This resulted in CoLeptin males having a significantly higher ejaculation frequency than MDLeptin males. There was no effect of either leptin treatment or MD on intromission frequency (Fig. 6e).

MD tended to decrease pseudo-ICI values [*F*(1,10) = 4.97; *p* = 0.05] with MD males having lower values than Co males (Fig. 6h). No effect of MD or leptin treatment was found on ICI (Fig. 6i). There was a significant interaction between MD and leptin treatment on copulatory rate [*F*(1,33) = 14.89; *p* < 0.005] with MDLeptin males having a lower rate than CoLeptin males (Fig. 6j).

Chi-squared analysis showed a significant decrease in the percentage of rats achieving ejaculation in the MDLeptin group (*χ*_1_ = 6.60, *p* < 0.05; Fig. 6l), with no effect on the percentage of rats displaying copulatory activity (Fig. 6k).

## Discussion

We previously reported that MD during 24 h at PND9–10 decreases adult body mass, energy intake, circulating leptin levels and increases the gain in body mass in response to a high-fat diet [13]. We speculated that at the least part of these metabolic effects were due to the dramatic decline in serum leptin levels during the separation period of MD [18], as modifications in neonatal leptin levels affect adult metabolism [28, 31–33]. Here, we found that some of the MD-induced modifications, including the increase in serum insulin and triglyceride levels and advanced external signs of pubertal onset in male rats and the decrease in serum triglyceride and estradiol levels in females, were reversed by neonatal leptin treatment, suggesting that part of the long-term effects of MD are mediated through changes in leptin levels. However, some effects, such as decreased body mass gain and food intake, were induced by leptin treatment, indicating a more complex interaction of these early events. We also found that MD did not result in the same metabolic modifications that we previously reported, such as significantly decreased body mass gain and circulating leptin levels in adulthood. These differences could possibly be derived from the daily handling of the pups in order to inject the control vehicle treatment.

### Body mass and food intake

Body mass was significantly decreased by MD until approximately PND57, which is in agreement with some of our previous studies where this decrease was found in adolescence and early adulthood, whereas in other studies, the decrease in body mass was found to persist into later adult life [13, 30, 34]. Neonatal leptin treatment further decreased body mass in MD rats, but there was no significant effect on the overall change in body mass during the study. In contrast, neonatal leptin treatment tended to increase adult body mass in control males and this was associated with a trend towards increased energy intake. This is in agreement with Toste et al. [35] where both body mass and food intake were increased in response to neonatal leptin treatment. However, other studies report no change in body mass [36–38]. These discrepancies may be due to the dose and timing of the leptin treatment or to other factors during development that can influence the response to leptin. Indeed, MD modified the response to neonatal leptin treatment in males as in these rats neonatal leptin treatment reduced weight gain and food intake. In control females, no effect of neonatal leptin treatment was observed on body mass or food intake and this is in agreement with a previous study Erkonen et al [37]. Moreover, in females, the response to neonatal leptin treatment was not modified by MD indicating that numerous factors, including sex, determine the long-term effects of changes in neonatal leptin levels on body mass and energy intake. Indeed, we have previously shown that neonatal leptin treatment decreases food intake in male rats, but this is a delayed effect [28]; thus, it is plausible that a change in food intake would be observed at older ages, at least in control males.

It is possible that body composition is affected by MD and/or the leptin treatment employed here and that this is not reflected in a change in body mass. Further studies are necessary to determine whether fat mass, as well as its distribution, is affected by MD and if this is due to the decline in circulating leptin levels induced by this experimental manipulation.

### Circulating hormone levels and glycemia

Here, we found males to have higher circulating leptin levels than females at PND36, and this was also found when they reached adulthood [39]. This is in accordance with the previous studies showing a sex difference in leptin levels [13, 19, 40], while here, this sex difference was observed even during the peripubertal period. In males, MD decreased leptin levels at PND36, but in adulthood, there was no effect of MD in either sex [39]. This is in contrast to our previous studies where MD was found to decrease serum leptin levels even in adulthood [18, 30, 34]. The stress of saline injection is reported to affect leptin levels [41], and this could mask the effect of MD possibly by even modulating circulating leptin levels in the vehicle-treated rats. Neonatal leptin treatment is reported to increase or have no effect on circulating leptin levels in later life [28, 37, 41]. Moreover, blocking the neonatal leptin surge was found to have no effect on body mass or leptin levels during the peripubertal period [42], but was found to decrease adult body mass and leptin levels [28]. Thus, as with body mass, the long-term effects on circulating leptin levels most likely depend on how and when the neonatal leptin surge is manipulated, as well as other neonatal conditions such as stress.

In both MD male and female rats, the AUC during the OGTT was decreased, with no effect of leptin treatment. During the process of MD, glycemia is decreased [18] and whether this underlies the long-term effect on glucose metabolism deserves to be analyzed. There was no long-term effect of MD on fasting glycemia, but fasting insulin levels were increased in MD males, which might suggest a degree of insulin insensitivity. This is in contrast to our previous studies [13, 18], again suggesting that handling of MD rats during vehicle injections could affect some outcomes of the MD procedure. In control males treated with leptin, insulin levels were increased, which is in concordance with Passos et al. [43] who reported that young rats treated with leptin during early postnatal life become hyperinsulinemic. However, the combination of MD and leptin treatment returned fasting insulin levels to that of controls.

We have previously shown that in females, MD reduces corticosterone levels in adolescence [26, 44]. Here, we found corticosterone levels to be reduced by MD at PND36 in both males and females, with leptin treatment normalizing this effect. However, at PND90 corticosterone levels were unaffected by MD or leptin treatment, although they tended to be reduced by MD in females with leptin treatment having no effect at this age. Thus, the effects of MD and leptin on circulating corticosterone levels appear to be both age and sex dependent. Moreover, at both ages, circulating corticosterone levels were higher in females compared to males, as previously reported [18, 45].

### Hypothalamic neuropeptides

Males that were exposed to MD appear to be more susceptible to the effect of neonatal leptin on food intake, with a reduction in accumulated energy intake, and this reduction could be related to the increase in hypothalamic mRNA levels of the anorexigenic neuropeptide POMC. However, increased POMC mRNA levels were also found in leptin-treated controls and MD vehicle-treated rats, where food intake and weight gain were not reduced. In addition, orexin levels were increased in MDLeptin males, which would be inclined to increase food intake. The mRNA levels of this orexigenic neuropeptide were increased in leptin-treated controls of both sexes, although, food intake was not affected. Thus, the changes in the mRNA levels of metabolic neuropeptides did not correlate with modifications in food intake or weight gain. Further studies are necessary to determine how MD and early leptin treatment modify peptide levels and secretion of these neuropeptides, including the synaptic organization of these metabolic circuits. The increase in POMC mRNA levels in MD males is discordant with previous results where we found no effect of MD on hypothalamic mRNA levels of POMC, CART, NPY, or AgRP at different post-natal ages [13, 18].

### Appearance of external signs of pubertal onset

Metabolic modifications during early life, especially increased weight gain, are suggested to impact on pubertal development with changes in leptin levels possibly being involved in this phenomenon [46–49]. Here, we found that both neonatal leptin treatment and MD advanced the appearance of the external signs of puberty in control male rats. In contrast, pubertal onset was delayed by leptin treatment in both control and MD females, with no effect of MD alone.

Leptin is considered to be a permissive factor for puberty [50], and increased fat mass and leptin levels are thought to advance pubertal onset [51]. However, this early increase in leptin levels delayed pubertal onset in females. It is possible that the age at which the subject is exposed to increased leptin levels determines how pubertal development is affected. Although hypothalamic expression of members of the kisspeptin system was unaffected, the delay in pubertal onset could be due to modifications in this system that do not involve changes in mRNA levels, such as synaptic connectivity or secretion patterns. Likewise, although there was no effect of either MD or leptin treatment on pituitary βLH or βFSH mRNA levels, the pattern of gonadotropin release could be affected and this could in turn affect the activation of the reproductive axis. In males, there was an increase in hypothalamic CART mRNA levels. This neuropeptide is involved not only in metabolism, but also in other physiological functions such as stress or reproduction, as CART can facilitate leptin’s action on GnRH neurons to influence pubertal onset [52]. However, it should be taken into consideration that the expression levels of neuropeptides and gonadotropins were measured in adult animals, and this may not reflect on what occurs during the pubertal transition. Further studies are necessary to determine what occurs at this specific time-point.

### Sex steroids and sexual behavior

Early environmental stresses, such as neonatal handling or perinatal maternal food restriction, can have long-term consequences on the reproductive axis and sexual behavior in both males and females [53–57]. We previously reported that circulating testosterone levels are decreased by MD [30], while here, this decline did not reach significance. Neonatal leptin treatment increased testosterone levels in adulthood, but only in MD males. Thus, there was an interaction between MD and leptin treatment with the combination of these treatments resulting in increased circulating testosterone levels.

Leptin regulation of normal male reproduction includes both central and testicular actions [58, 59], but no effect of either MD or leptin treatment was found on the expression of gonadotropins or neuropeptides involved in reproduction. As stated above, it is possible that the neuronal connectivity and/or hormonal pulsatility of this system are modified, with subsequent effects on physiological functions. Leptin also has a direct inhibitory action on testicular steroidogenesis [59, 60], which has been proposed as the link between decreased testosterone secretion and hyperleptinemia in obese men [59, 61]; thus, neonatal leptin treatment could possibly have a direct effect on testicular development. Indeed, we have recently shown that blockage of the neonatal leptin surge increased testicular volume in perinatal males [42]. The results reported here suggest that there is an interaction between early neonatal stress, such as MD, and changes in leptin levels on serum testosterone concentrations.

We analyzed whether the changes in testosterone levels were associated with modifications in sexual behavior. To date, there is no information in the literature as to how MD or neonatal leptin levels affect sexual behavior. In rat models of sexual behavior, mount latency, mount frequency, and intromission latency can be considered as measures of sexual desire and motivation, while intromission and ejaculation latency are measures of performance (consummatory behavior) [62]. Leptin treatment of control males appears to affect sexual performance in control males, decreasing ejaculation latency, but not in MD males. However, although leptin treatment of MD males increased serum testosterone levels, it decreased sexual motivation. The only apparent effect of MD alone on sexual behavior was a decrease in pseudo-ICI, which might be interpreted as an increase in sexual desire and motivation. Libido is under psychosomatic, neurogenic, vascular, and hormonal control (Waller et al. 1985). Current hypotheses suggest that dopaminergic activity is associated with sexual desire and erectile response, while central serotonergic activity is inhibitory to the erectile and orgasmic function [63]. In line with this, in previous studies, we found an increase in serotonin levels due to MD [64]. Thus, the MD-related increase of pseudo-ICI might be more associated with an increase of sexual desire rather than consummatory behavior. However, neonatal leptin treatment might function to increase sexual performance, as shown by its effects on ejaculation latency and mount/intromission frequency.

In females, estradiol levels were decreased by MD and returned to control levels with leptin treatment. Leptin plays an important role in female reproduction, with effects on gonadal function, pregnancy, and implantation [65–67], with female fertility being dependent on adequate metabolic status [68, 69]. Deregulation of leptin levels might be responsible for some reproductive problems such as infertility [70]; however, little is known regarding long-term effects of early stress and modifications in leptin levels on female reproduction. It is possible that the dramatic reduction in leptin produced by MD is interpreted as a signal that the nutritional status of the immediate environment is not conducive to future reproduction. The possible involvement of leptin in this phenomenon is suggested by the fact that neonatal leptin treatment normalized adult estradiol levels. Whether MD affects female fertility remains to be determined.

### Sex differences in the response to MD and neonatal leptin treatment

Males and females differ in their response to MD and neonatal leptin treatment, as well as the interaction between these two factors. As the development of the male and female brain differ temporally [71], the response to these experimental manipulations might be expected to differ. Indeed, the modifications in cell turnover and neurotrophic factors during MD are different between males and females [18], suggesting a differential response to this stress. Moreover, the neuroendocrine systems studied here are sexually dimorphic in normal adult animals [72]; hence, one might expect the long-term responses to this early stress to also differ. In general, females appear to be less affected in the studies presented here, which could indicate a greater resilience to factors that affect growth and reproduction.

### Summary and critical discussion

In summary, the results reported here further emphasize the importance of the postnatal leptin surge in brain development and indicate that the long term-consequences depend upon the manner in which this disruption occurs, as well as the sex of the animal. Indeed, some of the consequences of MD appear to be due, at least in part, to modifications in the leptin surge as leptin treatment of MD rats normalized some parameters. However, in some instances, MD and leptin treatment had the same effect or even additive effects. One caveat that must be considered is the regimen of leptin treatment. The daily injection results in a supraphysiological peak of serum leptin levels by 1 h after treatment. These levels remain elevated at 3 h and began to decline at 6 h after injection, returning to baseline levels by 24 h [28]. This leptin regimen induced long-term effects even in control rats. However, the response to this leptin treatment varied depending on the baseline circumstances of the animal (MD or control) or whether they were male or female.

Another caveat that should be taken into consideration when analyzing the data presented here is that we have used more than one rat from each individual litter. Although the statistical analyses indicated that there was no litter effect in these studies and litters were equalized in the number of pups and balanced in the number of males and females, the response of offspring to changes in leptin levels during development can be affected by maternal/litter characteristics [38].

The experimental procedure of maternal deprivation not only modifies circulating leptin levels, but it also affects the levels of other hormones, such as corticosterone and insulin, and affects maternal behavior once the pup is returned to its mother [34]. This increase in maternal care may buffer or compensate the negative consequences of the extended mother/pup separations [73]. Moreover, the changes in diverse factors most likely interact to induce some of the diverse long-term outcomes of MD. Indeed, the experimental procedure of MD has been extensively used as a model of neuropsychiatric symptoms, but it also has been shown to affect the immune and endocrine systems [74]. Similar situations may also occur in human development. For example, in situations of child neglect, more than one factor is affected (*e.g*.*,* stress, food intake, and possibly temperature) and there are long-term effects on numerous systems [75, 76]. However, as changes in neonatal leptin levels are known to affect the development of some endocrine systems [8], here, we asked whether the MD-induced effects on metabolism and reproduction could be explained, at least in part, by the resulting modifications in leptin levels. Indeed, although we are unable to separate the long-term effects of each individual change that occurs during MD or their interactions, our results indicate that the decrease in serum leptin levels induced by MD is involved in some of the observed effects on neuroendocrine systems.

## Conclusions

The results reported here clearly demonstrate that the early neonatal environment has important long-term influences on neuroendocrine systems, including puberty and sexual behavior, and that these outcomes are complex depending on numerous interacting factors including the sex of the animal. Changes in serum leptin levels during maternal deprivation appear to be involved in at least some of the resulting long-term metabolic and reproductive effects of this early stress. Moreover, our results further emphasize the importance of appropriate control groups during early manipulations due to the vulnerability of the developing systems even to vehicle treatments.

## Abbreviations

AgRP: agouti-related peptide
βFSH: follicle-stimulating hormone
βLH: luteinizing hormone
BDNF: brain-derived neurotrophic factor
BPS: balano preputial separation
CART: cocaine- and amphetamine-regulated transcript
Co: control, non-deprived
GFAP: glial fibrillary acidic protein
HTr: rostral hypothalamus
ICI: inter-copulatory interval
IL6: interleukin 6
LepR: leptin receptor
MBH: caudal or medial basal hypothalamus
MD: maternal deprivation
NCAM: neural cell adhesion molecule
NPY: neuropeptide Y
OGTT: oral glucose tolerance test
PND: postnatal day
POMC: proopiomelanocortin
Pseudo-ICI: pseudo inter-copulatory interval
TNFα: tumor necrosis factor alpha
Vh: vehicle
VO: vaginal opening
